# Highly suspected primary intraocular lymphoma in a patient with rheumatoid arthritis treated with etanercept: a case report

**DOI:** 10.1186/s12886-018-0832-0

**Published:** 2018-06-28

**Authors:** Woo Keun Song, Ah Ran Cho, Young Hee Yoon

**Affiliations:** Department of Ophthalmology, College of Medicine, University of Ulsan, Asan Medical Center, 88 Olympic-ro 43-Gil, Songpa-gu, Seoul, 05505 South Korea

**Keywords:** Primary intraocular lymphoma, Etanercept, Methotrexate, Spectralis-domain optical coherence tomography

## Abstract

**Background:**

To describe a case of highly suspected primary intraocular lymphoma (PIOL) in a patient using etanercept for the treatment of rheumatoid arthritis.

**Case presentation:**

A 50-year-old female patient presented with decreased vision in her left eye that lasted for a week. She had a 15-year history of seropositive rheumatoid arthritis (RA), and had been taking weekly etanercept for the preceding 8 months. Funduscopic examination and SD-OCT showed a swollen ellipsoid zone (EZ) and a retinal pigment epithelium (RPE) irregularity of the right eye. We also noted EZ disruption and a RPE irregularity in the left eye. As subretinal infiltration was aggravated in the right eye after the initial treatment, we completed a vitrectomy. Vitreous cytology revealed PIOL with positive CD20 immunostaining. She was treated with serial intravitreal methotrexate injections and systemic chemotherapy. After the treatment, subretinal infiltration and subRPE deposits were decreased in the right eye with no evidence of recurrence in either eye.

**Conclusions:**

This case suggests a potential relationship between immunosuppression with anti-TNFα medication, and increased risk for lymphoma, especially in patients with underlying rheumatologic disorders and especially in patients with suspected chronic refractory uveitis.

## Background

Tumor necrosis factor alpha (TNFα) plays a role in the normal inflammatory response and immune reaction. Although the safety and efficacy of anti-TNFα agents have already been established, the risk of lymphoma appears higher in patients treated with immune-modulating agents [1, 2]. This case report describes a patient with highly suspected primary intraocular lymphoma (PIOL), possibly associated with use of the approved anti-TNFα agent etanercept for treatment of rheumatoid arthritis (RA) [3].

## Case presentation

A 50-year-old female patient presented with a one-week history of decreased vision in her left eye. She had a 15-year history of seropositive RA treated with methotrexate and deflazacort. Because of an unsatisfactory response to those regimens, she was initiated on 25 mg per week of etanercept 8 months prior to presentation.

**Fig. 1:**
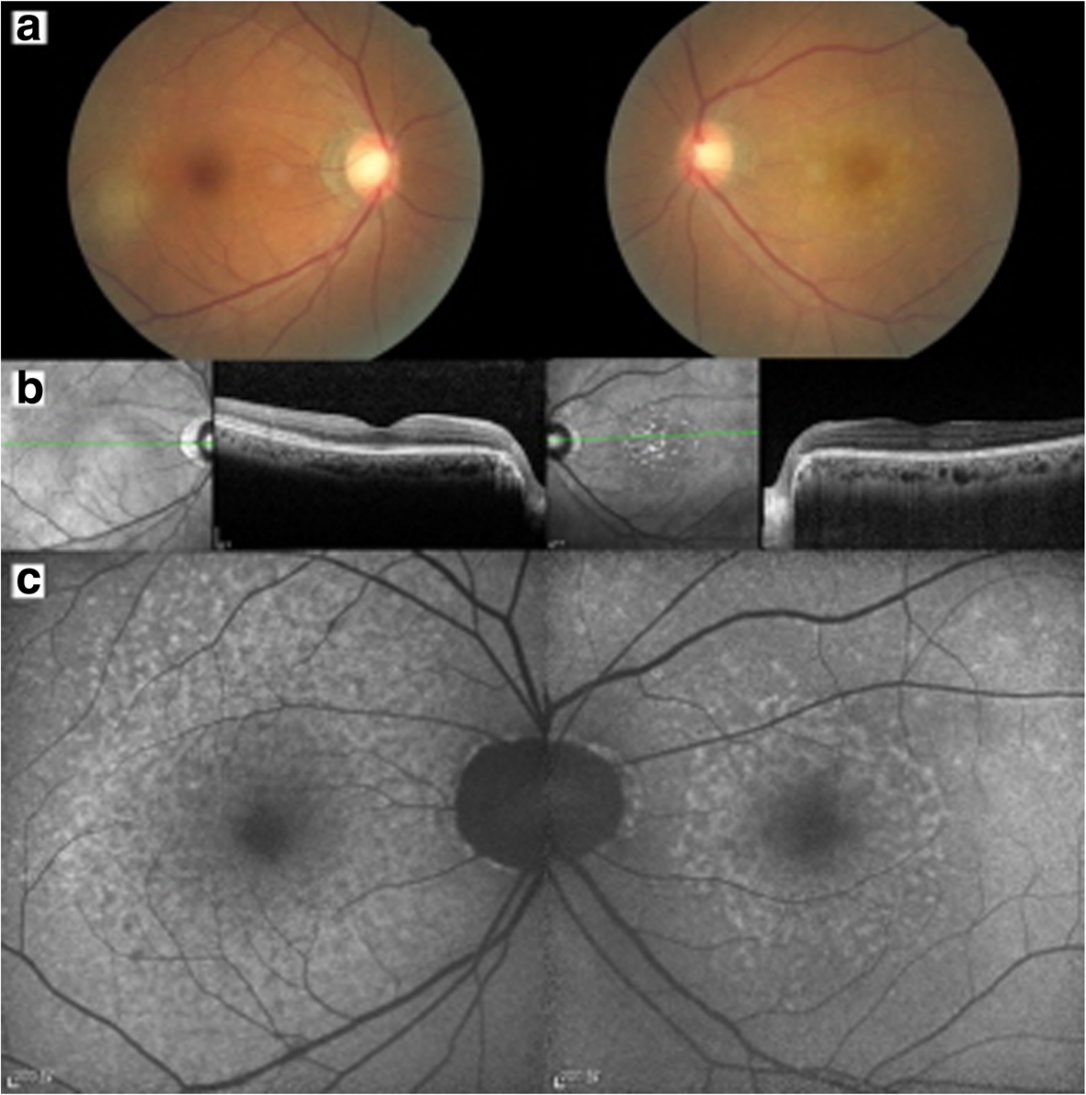
Initial ophthalmologic findings. **a** Funduscopic examination showed a granular infiltration temporal to the macula in the right eye and in the whole macular area of the left eye. **b** Spectral-domain optical coherence tomography showed a swollen ellipsoid zone and a retinal pigment epithelium irregularity in the right eye and an ellipsoid zone disruption in the left eye. **c** Fundus autofluorescence showed parafoveal granular hyperautofluorescence in both eyes

On presentation, her best-corrected visual acuity (BCVA) was 0.8 OD and 0.1 OS. There were no cells in the anterior chamber of either eye. Funduscopic examination showed granular infiltration at the temporal macula in the right eye and the foveal area in the left eye (Fig. 1a). Spectral-domain optical coherence tomography showed a swollen ellipsoid zone and retinal pigment epithelium (RPE) irregularities in the right eye and an ellipsoid zone disruption and RPE irregularity in the left eye (Fig. 1b). Fundus autofluorescence showed parafoveal granular hyperautofluorescence in both eyes (Fig. 1c).

**Fig. 2:**
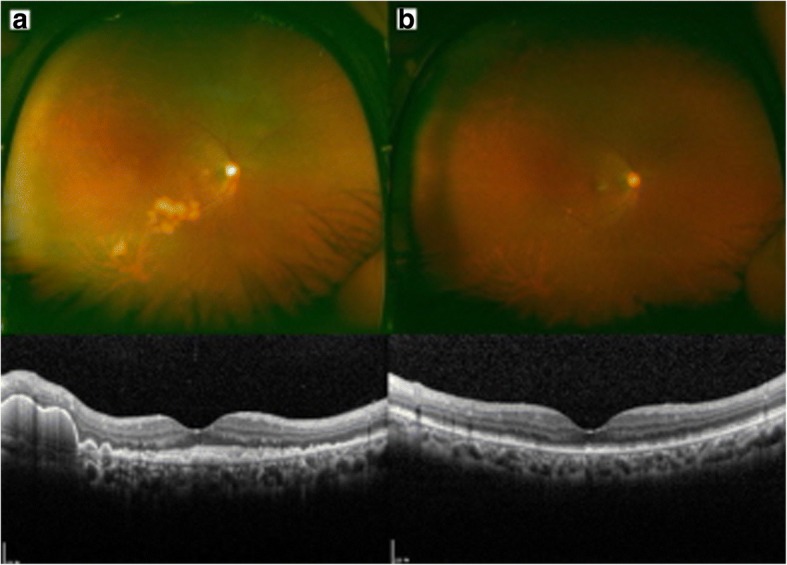
Follow-up ophthalmologic findings in the right eye. **a** Two months after steroid treatment, yellow-white subretinal deposits had developed. The ellipsoid zone swelling area extended to the subfoveal area, and sub-retinal pigment epithelium (RPE) deposits were also increased. **b** Six months following intravitreal methotrexate treatment, subretinal and sub-RPE infiltration decreased, resulting in severe photoreceptor disruption

Based on the negative results of various blood tests, she was diagnosed with noninfectious uveitis and started on oral prednisolone. She noticed mild improvement 1 month after treatment, but 2 months after treatment she showed a visual loss to 0.1 in her right eye. Compared with the initial visit, the swollen ellipsoid zone area extended under the fovea and subRPE yellow-white deposits were newly developed in the inferotemporal area (Fig. 2a). Also, mild vitreous opacity with haziness was noticed in the right eye. We suspected primary intraocular lymphoma (PIOL), and a 25-gauge microincision vitrectomy in right eye was performed. Vitreous cytology revealed atypical mononuclear cells with positive CD20 immunostaining. PCR of the vitreous fluid was negative for herpes and cytomegalovirus.

The patient was evaluated by an oncologist, and no lymphoma involvement in the brain was found. She was treated with high-dose systemic methotrexate as well as intravitreal methotrexate injections (400 μg/0.1 mL) twice weekly for 4 weeks for induction, once weekly for 8 weeks, and once monthly for 9 months in the right eye. Given the potential causal relationship between PIOL and anti-TNFα agents, etanercept was discontinued.

Three months after treatment, her right eye showed a decrease in subretinal infiltration and subRPE deposits, which further resolved at 6 months following treatment (Fig. 2b). Over the course, her left eye also showed gradual improvement in funduscopic examination as well as SD-OCT findings without intravitreal methotrexate injection, potentially due to systemic chemotherapy. Because of severe photoreceptor disruption, BCVA was 0.1 OD and 0.4 OS at the final visit.

## Discussion

The risk of lymphoma is still a major concern in patients using anti-TNFα agents. The exact mechanisms underlying lymphomagenesis are unclear. However, considering several anticancer mechanisms of TNF, such as induction of tumor vessel obstruction and tumor immune surveillance, effects related to anti-TNFα agents may contribute to tumor development by suppressing the anticancer mechanisms of TNF [4].

Reported rates of cytological sensitivity and specificity for the diagnosis of primary vitreoretinal lymphoma (PVRL) vary widely, but cytology alone is able to confirm PVRL in 45–60% of cases, and false positive results are considered rare. Experienced technicians and ocular cytopathologists play a major role in this success rate [5, 6]. In addition to the cytopathologic diagnosis, multicolor flow cytometry methods such as monoclonality can support B or T cell PVRL [7]. Either a B-cell population stains positively for B-cell markers (CD19, CD20, or CD22) with restricted expression of either κ or λ, or a T-cell population stains positively for T-cell markers (CD3, CD4). Furthermore, cytokine analysis of IL-10 and IL6 ratios greater than 1.0 are suggested diagnostic criteria for lymphoma [8–10]. Also, methods for detecting IgH gene rearrangement can serve as an additional diagnostic method [11, 12]. In this case, because we lacked a laboratory setting, cytopathologic diagnosis was made microscopically. We noted atypical cytomorphology with CD20 positivity, suggestive of lymphoma. The gold standard is pathologic diagnosis for lymphoma, and pathologic confirmation was made through an experienced pathologist.

Because no standard therapy for PVRL has been established, the International Primary CNS Lymphoma Collaborative Group organized a symposium on PVRL in 2011. Their guidelines established local treatment [intra-vitreal methotrexate, intravitreal rituximab, or low-dose (30–35 Gy) stereotactic external beam radiotherapy to the eye] with close follow as the preferred treatment option for cases without CNS and systemic involvement. If both eyes are involved, systemic chemotherapy has been suggested in addition to intravitreal medications for bilateral PVRL [13, 14]. Clinically, pars plana vitrectomy is frequently performed for PVRL biopsy because its diagnostic yield is superior to that of vitreous aspirate. A 25-gauge transconjunctival sutureless vitrectomy is a safe and effective technique for diagnosing PVRL. Considering the recent trend of minimally invasive vitrectomy, 25-gauge vitrectomy is an appropriate diagnostic method for vitreous sampling and diagnosis [15, 16]. In this case, suspicious bilateral involvement of PIOL, without CNS involvment with acutely developed in the unilateral eye, advocates the treatment of systemic chemotherapy treatment with intravitreal methotrexate injection and 25-gauge vitrectomy for diagnostic purposes.

Despite numerous systematic reviews and meta-analyses, there is insufficient evidence to establish a causal relationship between use of anti-TNFα agents and lymphomagenesis. The overall malignancy rate of anti-TNFα agents is reportedly 0.6 per 100 person-years, and the rate of etanercept is 10.47 per 1000 person-years [17]. The use of biologics among patients with RA, included in randomized controlled trials of at least 6 months’ duration, was not significantly associated with an increased risk of malignancy compared with other disease-modifying antirheumatic drugs, or compared to placebo [18]. In another study, there was no difference in the risk of lymphoma for the TNF inhibitor versus the biological-naive group: hazard ratio (HR) 1.00 (95% CI 0.56 to 1.80). No differences in risk were observed for individual TNF inhibitors [19, 20]. There is no evidence that tumor necrosis factor inhibition influences the risk of lymphoma, over background risk, in subjects with RA. However, there are case reports of primary intraocular B-cell lymphoma arising during methotrexate and tumor necrosis factor inhibitor treatment [21]. A direct relationship between primary intraocular lymphoma or PVRL and the use of biologic agents remains controversial; however, clinicians should be aware of the possibility of PIOL in patients with chronic refractory uveitis.

Our patient could represent a case of primary intraocular lymphoma, possibly related to the use of the anti-TNFα agent etanercept. A diagnosis of intraocular lymphoma should be suspected when patients with underlying rheumatologic disease develop chronic refractory uveitis during anti-TNFα treatment.

## Conclusions

Ophthalmologists should be aware of the relationship between immunosuppression via anti-TNFα medication, and an increased risk for lymphoma, especially in patients with underlying rheumatologic disorders, and especially in cases with suspected chronic refractory uveitis. Further evidence is needed pertaining to cancer risk in patients who are using, or have used, anti-TNFα agents.

## Abbreviations

BCVA: Best corrected visual acuity
EZ: Ellipsoid zone
PCR: Polymerase chain reaction
PIOL: Primary intraocular lymphoma
PVRL: Primary vitreoretinal lymphoma
RA: Rheumatoid arthritis
RPE: Retinal pigment epithelium
SD-OCT: Spectralis-domain optical coherence tomography
TNF: Tumor necrosis factor
